# Stomatal and growth responses to hydraulic and chemical changes induced by progressive soil drying

**DOI:** 10.1093/jxb/erx381

**Published:** 2017-11-06

**Authors:** Xiaoqing Li, Sally Wilkinson, Jianbo Shen, Brian G Forde, William J Davies

**Affiliations:** 1 Lancaster Environment Centre, Lancaster University, UK; 2 Key Laboratory of Plant-Soil Interactions, Department of Plant Nutrition, Ministry of Education, China Agricultural University, China

**Keywords:** Abscisic acid (ABA), drought, ethylene, hormone, maize, physiological responses, root, shoot

## Abstract

A better understanding of physiological responses of crops to drought stress is important for ensuring sustained crop productivity under climate change. Here, we studied the effect on 15-day-old maize (*Zea mays* L.) plants of a 6 d non-lethal period of soil drying [soil water potential (SWP) decreased from –0.20 MPa to –0.81 MPa]. Root growth was initially stimulated during drying (when SWP decreased from –0.31 MPa to –0.38 MPa, compared with –0.29 MPa in well-watered pots), followed by inhibition during Days 5–6 (SWP from –0.63 MPa to –0.81 MPa). Abscisic acid (ABA) in the root began to accumulate as the root water potential declined during Days 2–3. Leaf elongation was inhibited from Day 4 (SWP less than –0.51 MPa), just after leaf ABA content began to increase, but coinciding with a decline in leaf water potential. The stomatal conductance was restricted earlier in the younger leaf (fourth) (on Day 3) than in the older leaf (third). The ethylene content of leaves and roots decreased during drying, but after the respective increase in ABA contents. This work identified critical timing of hydraulic and chemical changes at the onset of soil drying, which can be important in initiating early stomatal and growth responses to drought.

## Teaser

This study synchronously investigated maize growth and physiological responses to progressive soil drying. It indicates that hydraulic and chemical changes may regulate plant development and functioning during onset of drought.

## Introduction

Drought is a major factor restricting crop production in many regions of the world (Boyer, 1982; Boyer *et al.*, 2013). Whilst maize (*Zea mays* L.) is among the top three staple crops worldwide (Varshney *et al.*, 2012), its production is likely to suffer more from drought stress in the future under a changing climate with increased risk of high temperatures and more variable precipitation (Battisti and Naylor, 2009; Tardieu, 2012; Challinor *et al.*, 2014). Therefore, it is important to breed plants that are more drought resistant and to improve current irrigation management for agricultural systems. Both of these requirements can depend upon a better understanding of the physiological responses to drought stress of shoots and roots (Tuberosa *et al.*, 2007).

Unfortunately the term ‘drought’, as used in agriculture, is imprecise and does not have a universal definition (Wilhite and Glantz, 1985; Gilbert and Medina, 2016; McDaniel *et al.*, 2017). However, it is valuable to use a combination of indices to characterize a specific drought stress event (e.g. onset, severity, and duration), which can facilitate comparison and interpretation of specific plant drought responses (Lawlor, 2013). A non-lethal drought stress is common in the field and is considered to be an important target for the improvement of plant performance in droughted environments (Tuberosa *et al.*, 2007; Skirycz *et al.*, 2011).

Plants use different strategies to cope with different degrees of drought (avoidance and tolerance), including numerous responses to avoid water loss, continue water uptake at low soil moisture contents, or tolerate a low tissue water content, and thereby minimize the reduction of crop growth and yield under drought (Lawlor, 2013). These avoidance and tolerance strategies are accomplished through a range of physiological responses, such as reducing stomatal conductance and development of leaf area, changing root and shoot growth to enhance the root to shoot ratio, maintaining turgor pressure by reducing cellular solute potential (osmotic adjustment), etc. (Lawlor, 2013; Gilbert and Medina, 2016). Plant shoots and roots may respond differently to the same drought stress by means of development, growth, and other physiological changes (Munns and Cramer, 1996; Romero *et al.*, 2017; Zhang *et al.*, 2017). Shoot growth is generally more inhibited by drought than root growth (Sharp and Davies, 1979; Durand *et al.*, 2016). In some cases, under mild drought, root growth may be promoted by soil drying, which is of great importance in maintaining sufficient water supply for the plant (Sharp and Davies, 1979; Kano *et al.*, 2011). Westgate and Boyer (1985) showed that the maize nodal root could continue its elongation when the water potential in its growing region was –1.4 MPa, while the elongation of the stem, silks, and leaves from the same plant was completely inhibited when the water potentials in their growing regions were –0.50, –0.75, and –1.0 MPa, respectively. Similarly, the primary root elongation rates of maize, soybean, cotton, and squash were reduced but were maintained when the substrate water potential was –1.6 MPa, while the shoot growth was completely inhibited at –0.8 MPa (Sharp, 2002).

Phytohormones have been shown to regulate plant development and growth under drought stress (Santner *et al.*, 2009; Pierik and Testerink, 2014). The concentration of abscisic acid (ABA), one of the most important drought-relevant hormones, increases under drought stress in many plant species (e.g. Arabidopsis, maize, and potato) (Zhang and Davies, 1989; Huang *et al.*, 2008; Puértolas *et al.*, 2015). It is also suggested that the concentration of ABA in the root could be an indicator of a local change in soil water availability (Zhang and Davies, 1989). Furthermore, the accumulation of ABA under drought stress is reported to be responsible for stomatal closure and the inhibition of shoot and root growth (Chen *et al.*, 2013; Harris, 2015). Mild drought can stimulate root growth, while severe drought can inhibit it (Sharp and Davies, 1979; Creelman *et al.*, 1990). Accordingly, stimulatory and inhibitory effects on root growth were shown when ABA was applied to plants at low and high concentrations, respectively (Xu *et al.*, 2013; Li *et al.*, 2017).

Ethylene is a gaseous plant hormone, which is probably also involved in plant drought responses (Sharp and LeNoble, 2002; Kazan, 2015). Previous studies have indicated that drought stress may promote, restrict, or not affect the ethylene production in various plant species (Morgan *et al.*, 1990; Sharp and LeNoble, 2002; Arraes *et al.*, 2015). Morgan *et al.* (1990) reported that intact cotton and bean plants showed reduced ethylene production during slow soil drying, in contrast to the responses shown by detached leaves under rapid desiccation. Therefore, the types of drought stress and sampling methods could affect the ethylene production result. Ethylene has been shown to be an inhibitor of shoot growth, root elongation, and lateral root initiation (Pierik *et al.*, 2006; Muday *et al.*, 2012). A series of studies have suggested that significant accumulation of ABA is necessary to prevent extra ethylene production and thus ameliorate its inhibition of maize shoot and root growth under low water potentials (Saab *et al.*, 1990; Sharp and LeNoble, 2002). Hence, it has been assumed that the interaction between ABA and ethylene plays an important role in regulating plant drought response (Sharp and LeNoble, 2002; Tanaka *et al.*, 2005). Nevertheless, there is also good evidence for a controlling influence of plant hydraulics in the regulation of plant development and functioning under drought (e.g. Brodribb, 2009), and more precise estimation and measurement of intraorgan variation in hydraulic and chemical status of plant cells (e.g. Buckley *et al.*, 2017) highlights the difficulty of ruling in or out hydraulic and/or chemical control in individual studies. However, few studies have simultaneously investigated the gradual changes of hormone levels and leaf and root growth in response to a gradual soil drying, let alone the timing of these changes, which is prerequisite if we are to elucidate the complex signalling pathways which are important components of the plant drought response.

By subjecting 15-day-old maize plants to a 6 d non-lethal soil drying episode, the responses of leaf and root growth and physiological variables, such as endogenous ABA and ethylene accumulation, were investigated synchronously in this study. The results from this work imply the important involvement and the timing of hydraulic and hormonal changes in regulation of shoot and root growth during soil drying, and could provide useful plant physiological information for improving crop management under drought.

## Materials and methods

### Plant growth

The maize cultivar Earligold F1 (VSW041, Moles Seeds, UK) was used. In experiment one, 280 seeds (0.15–0.19 g per seed) were soaked in deionized water for 48 h and then pre-germinated on wet paper towels for 72 h in a controlled-environment (CE) room in the dark (temperature, 24 °C/18 °C; photoperiod, 14 h/10 h; relative humidity, 40%; light density, 350 μmol m^−2^ s^−1^). Then seedlings with a root length of 4–10 cm were transplanted into 155 pots (height, 24 cm; diameter, 6.4 cm; with stainless wire mesh at the bottom) with one seedling per pot. Each pot was filled with 785 g of moist soil (~628 g of dry soil) to make a 22 cm tall soil column. The soil was sieved (1 cm sieve) John Innes No.2 (Foremost, UK). After transplanting, each pot was watered thoroughly by adding 200 ml of water. Seedlings became visible on the next day and another 20 ml of water was added to each pot. The soil column was then drained for 1 h and weighed to determine the pot capacity for water (54% of soil water content, w/w soil dry weight). All pots were weighed and watered to the pot capacity every day until the 15th day, except on the seventh day after transplantation when 50 ml of Hoagland’s nutrient solution (pH 5.8–6.0) was given to each pot. The third leaf was expanded fully (the leaf collar became visible) by the 15th day after transplantation, which was set as the last watering day (Day 0) for the soil drying treatment.

One hundred and four plants at a similar growth stage were selected: 48 plants for the soil drying treatment and another 48 plants as the well-watered control during the following 6 d; in addition to these, 8 plants were sampled on Day 0 as the starting reference. Control plants were watered daily to pot capacity. Eight pots of each treatment were destructively harvested every day during Days 1–6. All of the pots were moved every other day to ensure a uniform growth environment.

This experiment was repeated once (experiment two). In experiment two, 170 seeds (0.15–0.19 g per seed) were pre-germinated and 95 seedlings were transplanted into pots. On the last watering day (the 15th day, Day 0), 65 plants at a similar growth stage were selected: 30 plants for each treatment (soil drying and well-watered) and 5 plants were sampled on Day 0. The growth condition and other processes in these two experiments were the same. Similar results were seen in these two experiments. The data presented here were combined results by treating every sample in either experiment as one replicate.

### Soil water content and soil water potential

**Fig. 1.**
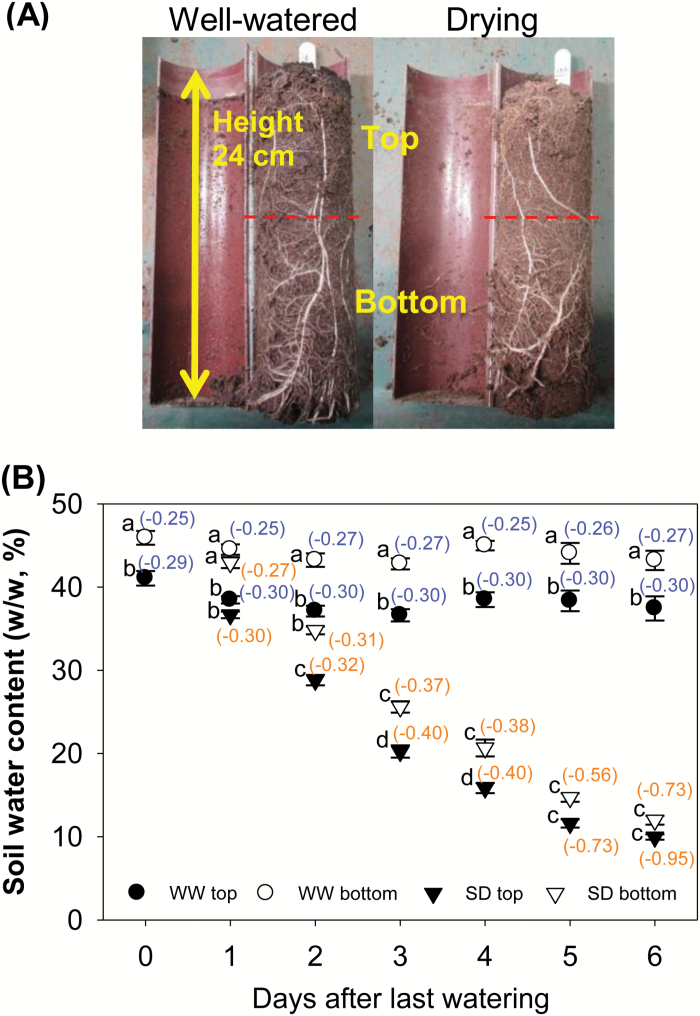
(A) Soil columns from the well-watered and soil drying treatments on Day 6 after the last watering; (B) soil water content in top and bottom parts of well-watered (WW) and soil drying (SD) treatments (Days 0–6). Pre-germinated maize seeds (Earligold F1) were transplanted into pots filled with sieved soil (John Innes No.2). Seedlings germinated from the soil surface after 1 d. All pots were weighed and watered to the pot capacity every day until the 15th day, except on the seventh day after transplantation when 50 ml of Hoagland’s nutrient solution (pH 5.8–6.0) were supplied to each pot. The third leaf was fully expanded on the 15th day after transplantation, and this day was set as the last watering day (Day 0). Plants at a similar growth stage were selected. The same experiments were conducted twice, and data presented here are the combined result. After Day 0, control plants were watered daily to pot capacity while watering ceased in the soil drying treatment for 6 d. Pots of each treatment were destructively harvested every day during Days 1–6. Each soil column was cut into top and bottom halves from the middle to measure the soil water content in the top and bottom parts. Points and bars are means ± SEs. Data were analysed using one-way ANOVA with Tukey’s *post-hoc* test, and different letters indicate a significant difference on the same day at *P*<0.05 (*n*=13 on Day 0 and *n*=9 on other days). Values in brackets are estimated soil water potentials (MPa) based on the soil water content values and the soil water characteristic curve (Supplementary Fig. S1).

After removing the shoot from the soil surface, the soil column was cut into top and bottom halves from the middle (Fig. 1A). After root tissue was removed, each part of the column was weighed (W_original_), oven-dried at 80 °C for about a week, and weighed again for dry weight (W_dry_). Then the soil water content (%, w/w) was calculated by [(W_orignial_−W_dry_)/W_dry_]×100%.

A soil water characteristic curve can be found in Supplementary Fig. S1 at *JXB* online. The soil water potential was measured by a thermocouple psychrometer (Wescor Inc., UT, USA) when the soil water content was >25% (water potential higher than –0.37 MPa) and by the WP4-T Dewpoint Potentiometer (Decagon Devices, WA, USA) when the water content was between 5% and 25%. The soil water potential result was estimated from this soil water characteristic curve based on soil water content values.

### Leaf elongation rate and root growth measurements

From the day before Day 0, the length of four growing leaves (the fourth to seventh leaves) was measured daily once visible. The leaf elongation rate (mm h^−1^) was calculated. After the incubation for root ethylene (see below), the entire root system was scanned and analysed for total root length and root surface area with the WinRHIZO Pro system (Regent Instruments Inc., Quebec, Canada). In each treatment, the mean of root length or surface area on the previous day was treated as the root length or surface area for that day for calculation of the daily increase in rates of these parameters (units: m d^−1^, cm^2^ d^−1^).

### Leaf and root water potential and solute potential

Leaf and root water potentials (Ψ_leaf_ and Ψ_root_) were measured with thermocouple psychrometers. Leaf discs (5 mm diameter) were punched from the middle of the third leaf (avoiding the midrib). The leaf disc was immediately wrapped in aluminium foil to minimize water loss and loaded into a C52 sample chamber (Wescor Inc.) within minutes for a 3 h incubation. The voltage was then recorded on a HR-33T Dew Point Microvolt meter (Wescor Inc.). The water potential in megapascals was converted from the recorded voltage based on the calibration with salt solutions of known osmotic potentials. A few roots (no root tips) were collected from the outer surface of the top two-thirds of the soil columns after the root tips were collected for ABA assay (see below). The roots were cut into small segments (5–8 mm). Ten to fifteen root segments were wrapped in aluminium foil and used to measure the water potential in the same way as for the leaf samples. During Days 0–6, leaf and root tissues were sampled from 10.00 h till 18.00 h in the light period of the CE room (06.00 h to 20.00 h) when a plant was destructively harvested on each day. Plants from well-watered and soil drying treatments were harvested alternately within each day (except Day 0).

The same leaf and root samples were then used to measure solute potentials (Ψ_s-leaf_ and Ψ_s-root_) by the same psychrometer. Samples were frozen by submergence into liquid nitrogen and then stored in a –20 °C freezer, defrosting before use. The voltage was record after 30 min incubation of samples and then converted to solute potential in megapascals. Leaf and root turgor pressures (Ψ_t-leaf_ and Ψ_t-root_) were then calculated for every sample according to the equation Ψ_t_=Ψ−Ψ_s_.

### Stomatal conductance

Stomatal conductance was measured daily between 07.00 h and 09.00 h (photoperiod started at 06.00 h) with an AP4 porometer (Delta-T Devices, Cambridge, UK). The third (fully expanded on Day 0) and the fourth (fully expanded on Day 2 or 3) leaves of each plant were measured. The measurement was on the abaxial leaf surfaces from both sides of the midrib in the middle one-third of each leaf. Two positions on each side of the midrib were measured, and the mean value of the four readings was used to represent the stomatal conductance for an individual plant.

### ABA assay for leaf and root tissues

In experiment one, the third leaves of every two of the eight plants from the same treatment were pooled as one replicate. In experiment two, the third leaf of each plant was treated as one replicate. The leaves were cut at the collars, folded into one 15 ml centrifuge tube, and immediately submerged in liquid nitrogen. Around 100 root tips (~3 cm) were collected from the top two-thirds of the soil column of the same two pots used for leaf sampling in experiment one. Similarly, ~40 root tips were collected from one plant in experiment two. The root tips were quickly washed with tap water, transferred into a 1.5 ml centrifuge tube, and submerged in liquid nitrogen. All samples were stored at –20 °C before being freeze-dried for 48 h. The samples were then ground, and ~30 mg of leaf tissue and all root tips were extracted with deionized water at 1:25 mg:μl ratio in a 1.5 ml centrifuge tube and shaken at 4 °C overnight. Then the competitive radioimmunoassay (Quarrie *et al.*, 1988) was used to determine ABA concentrations (ng g^−1^ DW). The extract was centrifuged at 12 000 *g* for 4 min and then 50 μl of supernatant was pipetted into the reaction buffer. This buffer contained 200 μl of 50% 50 mM phosphate-buffered saline (PBS) (pH 6.0), 100 μl of diluted antibody MAC 252, and 100 μl of diluted [^3^H]ABA. The mixture was then incubated for 45 min at 4 °C. The bound radioactivity of [^3^H]ABA was measured with a liquid scintillation counter (Packard TriCARB 1600TR liquid scintillation analyser, Canberra, CT, USA). A standard curve with eight ABA solutions [0, 62.5, 125, 250, 500, 1000, 2000, and 2 × 10^6^ pg 50 μl^−1^ (+)-ABA] was made from (±)-ABA (A1049, Sigma-Aldrich) and was measured with samples and used for calculating the ABA concentrations of samples.

### Ethylene release rates from leaf and root

In experiment one, four of the eight plants in each treatment were used for ethylene incubation every day during Days 1–6, while every plant was used in experiment two. The fifth leaf and the entire root system of a plant were used to quantify the ethylene release rate. The entire root system was washed out of the soil (within 30 min) after root tips were collected. Leaf and root samples were incubated in glass test tubes sealed with rubber stoppers for 1.5 h under light and dark, respectively. To prevent water loss from the sample, a piece of wet filter paper was enclosed. After the incubation, 1 ml of gas was taken with a syringe and injected into a GC system fitted with a flame ionization detector (FID; 6890N, Agilent Technologies, CA, USA) (Chen *et al.*, 2013). A 20 ppm ethylene/nitrogen standard gas (BOC Limited, Surrey, UK) was used to check the ethylene peak time and also for calibration. The leaf and root samples (after root scanning; see above) were oven dried and weighed. Then ethylene release rates (nl g^−1^ DW h^−1^) were calculated for leaves and roots.

### Statistical analysis

The statistical software SPSS 21.0 (IBM, USA) was used to perform either one-way ANOVA with Tukey’s *post-hoc* test or *t*-test at the *P*<0.05 level.

## Results

### Soil water content during soil drying

To establish a non-lethal progressive soil drying episode and to investigate maize root and shoot physiological responses during this process, several preliminary experiments were conducted and this 6 d drying treatment was chosen for this study. On the sixth day of soil drying, maize plants started to wilt, but this wilting phenomenon can be eliminated quickly by rewatering (data not shown). To determine the drought intensity of the soil drying treatment during the 6 d after the last watering, soil water contents of the top and bottom halves of soil columns were measured. The top half of the column had a lower soil water content than the bottom half of the column in both well-watered and drying treatments (Fig. 1B). The well-watered pots had a soil water content of 38% (soil water potential –0.30 MPa) and 44% (soil water potential –0.26 MPa) in the top and bottom soils on average during the 6 d, respectively (Fig. 1B). In contrast, the water content in the drying treatment declined from 37% (soil water potential –0.30 MPa) to 10% (soil water potential –0.95 MPa) in the top half soil and from 43% (soil water potential –0.27 MPa) to 12% (soil water potential –0.73 MPa) in the bottom half soil (Fig. 1B). Soil water contents in both the top and bottom halves of the drying treatment were significantly lower than those in the well-watered pots from Day 2 (Fig. 1B). The average water content of the soil columns in the drying treatment dropped gradually from pot capacity (54%, just after watering) on Day 0 to 11% on Day 6 (Fig. 1B), corresponding to water potentials of –0.20 MPa and –0.81 MPa, respectively (Fig. 1B; Supplementary Fig. S1).

### Effects of soil drying on leaf and root growth

**Fig. 2.**
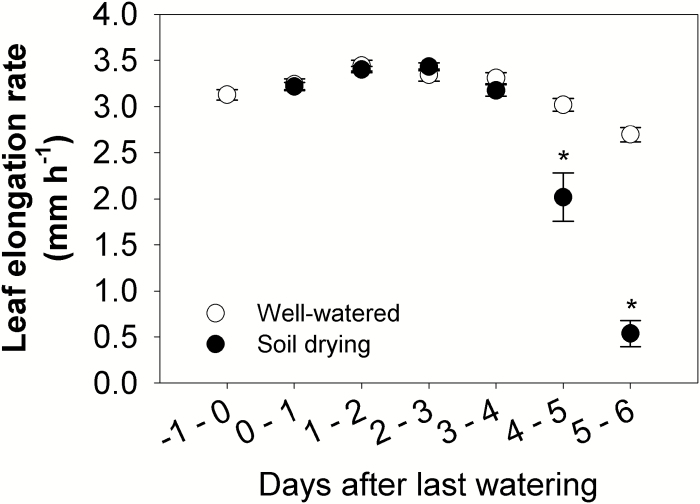
Leaf elongation rate of the fifth leaf of maize seedlings (the leaf was expanding and visible before the start of soil drying); *n*=13 replicates. Points and bars are means ± SEs. Data were analysed using the *t*-test, and asterisks indicate a significant difference between well-watered and soil drying treatments on the same day at *P*<0.05.

Maize leaf elongation rate, total root length, and total surface area were measured to indicate plant growth responses during soil drying. The results showed that soil drying significantly reduced the leaf elongation rate after Day 4 (the average soil water potential in drying pots was –0.51 MPa) (Figs 1B, 2; Supplementary Fig. S1). A >30% and ~80% reduction was seen, respectively, during Days 4–5 (the average soil water potential in drying pots decreased from –0.51 MPa to –0.63 MPa) and Days 5–6 (from –0.63 MPa to –0.81 MPa) (Figs 1B, 2; Supplementary Fig. S1). Other older (the fourth leaf) or younger leaves (the sixth and seventh leaves) showed a similar reduction in the elongation rate during soil drying (Supplementary Fig. S2).

**Fig. 3.**
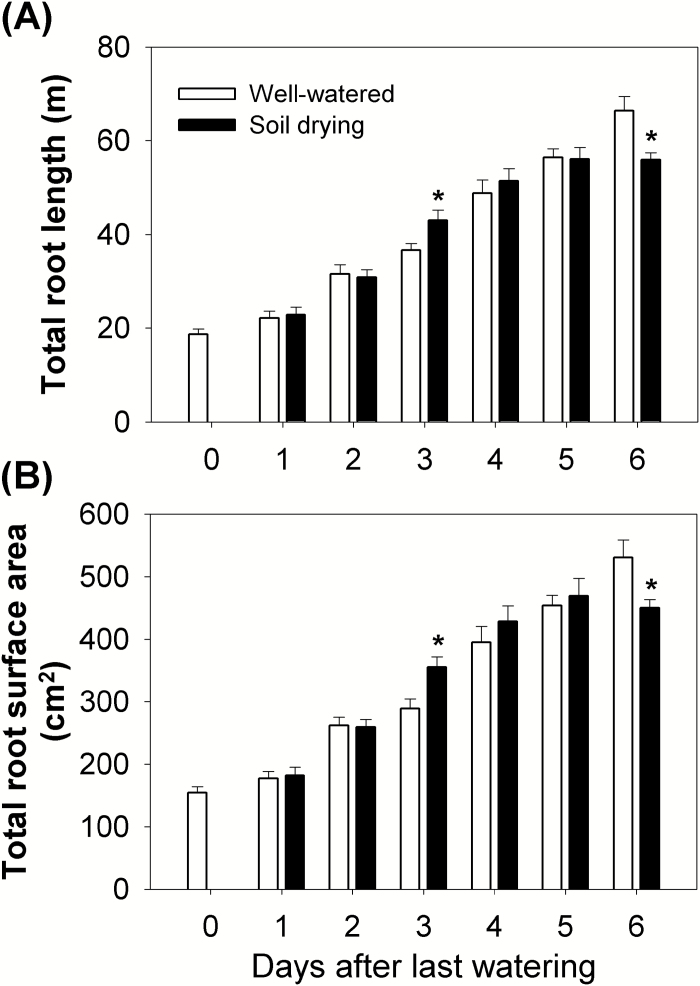
(A) Total root length and (B) total root surface area during the experimental period (Days 0–6). During the 6 d soil drying treatment (Fig. 1), the roots that were used for ethylene incubation in each treatment were scanned and analysed for total root length and root surface area using the WinRHIZO Pro system. Columns and bars are means ± SEs. Data were analysed using the *t*-test, and asterisks indicate a significant difference between well-watered and soil drying treatments on the same day at *P*<0.05 (*n*=9).

Maize in the soil drying treatment showed a larger total root length and surface area than the well-watered plants on Day 3 (the average soil water potential in drying pots was –0.38 MPa) (Figs 1B, 3; Supplementary Fig. S1), which was caused by a greater root growth rate during Days 2–3 (the average soil water potential in drying pots decreased from –0.31 MPa to –0.38 MPa) of the soil drying treatment, when drought was mild (Fig. 1B; Supplementary Figs S1, S3). However, maize subjected to the soil drying treatment had a smaller root system on Day 6 (the average soil water potential in drying pots was –0.81 MPa) (Figs 1B, 3; Supplementary Fig. S1), which was due to the reduced root growth rate after Day 3 when the drought became more severe (Supplementary Fig. S3).

### Physiological responses to soil drying

#### Changes in water potential and turgor pressure of leaf and root

**Fig. 4.**
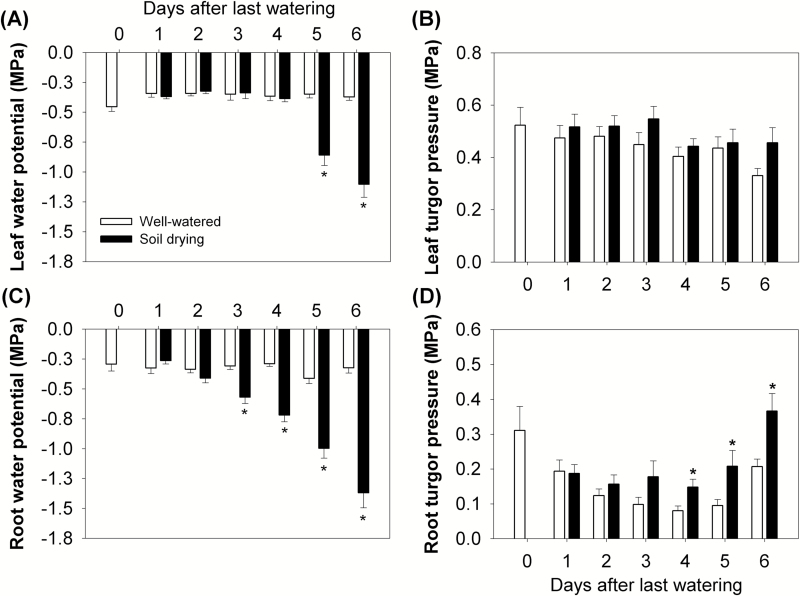
(A) Leaf water potential and (B) leaf turgor pressure of the third leaf during the experimental period (Days 0–6). (C) Root water potential and (D) root turgor pressure during the experimental period (Days 0–6). During the 6 d soil drying (Fig. 1), a leaf disc (5 mm diameter) from the middle of the third leaf (avoiding the midrib) or a root sample (10–15 root segments, 5–8 mm in length and without root tips) from the top two-thirds of the soil columns was incubated for 3 h in a C52 sample chamber in a thermocouple psychrometer. The voltage was then recorded on a HR-33T Dew Point Microvolt meter. The leaf and root samples were then frozen and thawed before they were used to measure the solute potentials, which were also measured by the same thermocouple psychrometer used for water potential measurement. Each sample was incubated for 30 min and the voltage was recorded. The voltage readings were then converted to water potentials and solute potentials, respectively. Columns and bars are means ± SEs. Data were analysed using the *t*-test, and asterisks indicate a significant difference between well-watered and soil drying treatments on the same day at *P*<0.05 (*n*=13).

Leaf water potential and solute potential of the third leaf were monitored as an indicator of leaf water status during soil drying. The leaf water potential in well-watered maize was between –0.34 MPa and –0.37 MPa during the 6 d period, while in the drying treatment it dropped to a significantly lower value on Day 5 (leaf water potential –0.86 MPa; the average soil water potential in drying pots was –0.63 MPa) and it decreased further to –1.10 MPa on Day 6 (Figs 1B, 4A; Supplementary Fig. S1). The leaf turgor pressure of both well-watered and droughted plants was lower than starting values of the respective treatments from Day 4 (Fig. 4B). However, the soil drying treatment did not reduce leaf turgor during the 6 d period when compared with controls (Fig. 4B).

The root water status was determined by measuring root water potential and calculating root turgor pressure. The root water potential was always around –0.30 MPa in the well-watered plants over the 6 d (Fig. 4C), which was close to the average soil water potential (Fig. 1B; Supplementary Fig. S1). In contrast, the root water potential in the soil drying treatment decreased from –0.26 MPa to –1.37 MPa between Day 1 and Day 6 (the average soil water potential in drying pots decreased from –0.29 MPa to –0.81 MPa) and was significantly lower than that in the well-watered plants from Day 3 (the average soil water potential in drying pots was –0.38 MPa) (Figs 1B, 4C; Supplementary Fig. S1). It is notable that the root water potential decreased along with, but remained lower than, the average soil water potential in the drying treatment from Day 2 (Figs 1B, 4C; Supplementary Fig. S1). Root turgor pressure was maintained and even increased in the treated plants over the 6 d (Fig. 4D), but was not significantly increased during the early stages of soil drying when increases in root growth were detected (Figs 3, 4D).

#### Changes in leaf stomatal conductance

**Fig. 5.**
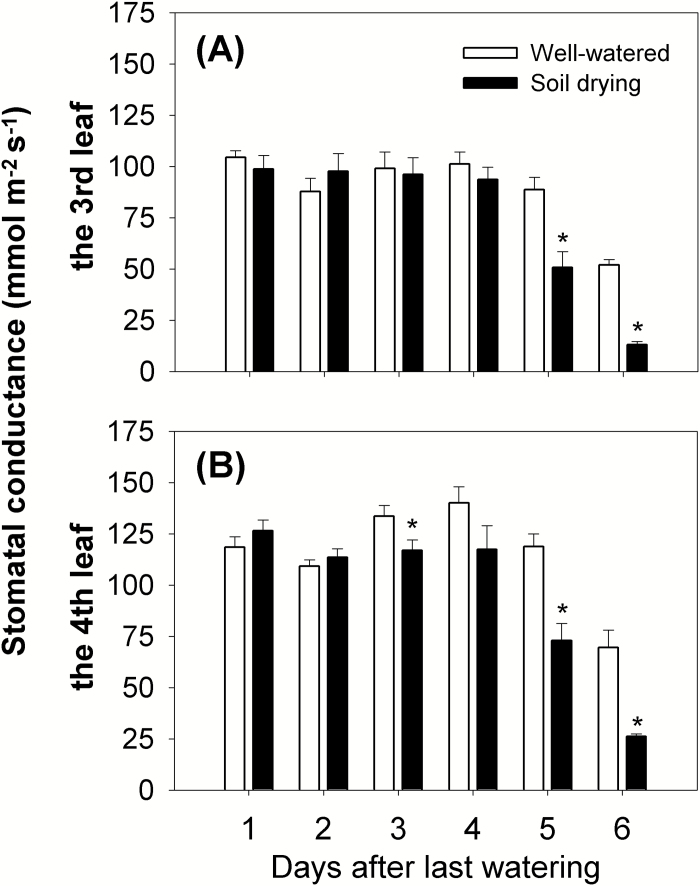
Leaf stomatal conductance of (A) the third leaf (the leaf was fully expanded before soil drying), (B) the fourth leaf (the leaf was fully expanded on Day 2 or 3) in response to soil drying. During the 6 d soil drying (Fig. 1), the third and fourth leaves of each plant were measured for stomatal conductance using an AP4 porometer. The measurement was on the abaxial leaf surface from both sides of the midrib in the middle one-third of each leaf. Two positions on each side of the midrib were measured, and the mean value of the four readings represented the stomatal conductance of the respective leaf. Columns and bars are means ± SEs. Data were analysed using the *t*-test, and asterisks indicate a significant difference between well-watered and soil drying treatments on the same day at *P*<0.05 (*n*=8).

The stomatal response to soil drying was monitored on a mature leaf (the third) and a younger one (the fourth). The stomatal conductance of the third leaf decreased along with soil drying from Day 5 (the average soil water potential in drying pots was –0.63 MPa) and decreased by 43% and 75% compared with the well-watered maize plants on Day 5 and 6, respectively (Figs 1B, 5A; Supplementary Fig. S1). However, the fourth leaf showed a higher stomatal conductance than the third leaf, by ~30% on average over the 6 d (Fig. 5). In addition, an earlier response of stomata to soil drying was seen in this younger leaf; a significant reduction in stomatal conductance (by 12%) was seen on Day 3 (the average soil water potential in drying pots was –0.38 MPa) in drying plants (Figs 1B, 5B; Supplementary Fig. S1). On the last 2 d of soil drying, the stomatal conductance in the fourth leaf decreased further (by 39% and 62%, respectively) (Fig. 5B).

#### Changes of ABA concentrations and ethylene release rates in leaf and root

**Fig. 6.**
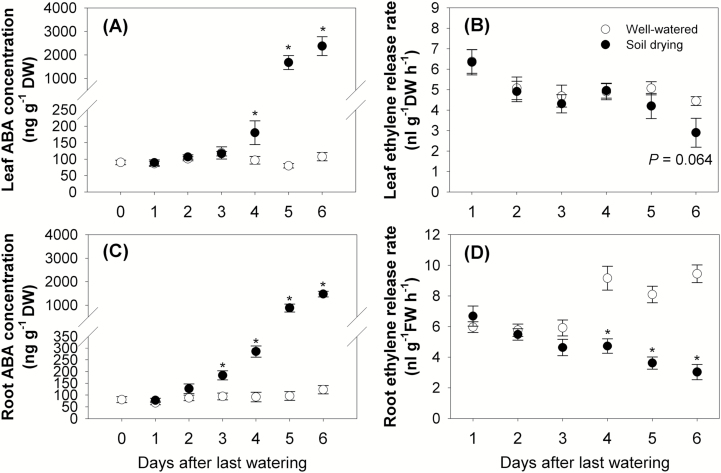
(A) Leaf ABA concentration in the third leaf (fully expanded before soil drying), (B) leaf ethylene release rate of the fifth leaf (expanding), (C) ABA concentrations in root tips, and (D) ethylene release rate of the entire root system. During the 6 d soil drying (Fig. 1), leaf samples were cut at the collars, and root tips (~3 cm each) were collected from the top two-thirds of the soil column. These samples were submerged in liquid nitrogen immediately and then stored at −20 °C before being freeze-dried for 48 h. Dry samples were ground and extracted with water and the extract was then used to determine the ABA concentration by radioimmunoassay. The fifth leaf was cut from the soil surface and then incubated for 1.5 h (under light in the CE room) with a piece of wet filter paper in a sealed glass tube. A whole root system of a plant was then washed and incubated similarly to the leaf sample but in the dark. Then 1 ml of gas was taken with a syringe and measured with a GC system fitted with a FID. The leaf or root sample was then oven dried for dry weight and the ethylene release rate was calculated. Points and bars are means ± SEs. Data were analysed using the *t*-test, and asterisks indicate a significant difference between well-watered and soil drying treatments on the same day at *P*<0.05 (*n*=9).

During the 6 d of the experiment, ABA concentrations in the third leaf of well-watered plants ranged between 80 ng g^−1^ DW and 119 ng g^−1^ DW (Fig. 6A), while in the soil drying treatment the concentrations increased to around twice this value on Day 4 (the average soil water potential in drying pots was –0.51 MPa) and >20 times this value from Day 5 (the average soil water potential in drying pots was –0.63 MPa) (Figs 1B, 6A; Supplementary Fig. S1). In contrast, the ethylene release rate of the fifth leaf only showed a reduction with soil drying treatment on Day 6 (by 35%, *P*=0.064; the average soil water potential in drying pots was –0.81 MPa) (Figs 1B, 6B; Supplementary Fig. S1). In one preliminary 5 d soil drying experiment, ethylene release rates of the fifth and sixth leaves showed a significant reduction during soil drying from Day 4, which was 1 d later than the increase of leaf ABA concentration (Supplementary Table S1; Supplementary Fig. S4).

The ABA concentration in the root tips of well-watered maize ranged between 66 ng g^−1^ DW and 123 ng g^−1^ DW, which was similar to ABA concentrations in the third leaf (Fig. 6A, C). In response to soil drying, the ABA concentration in root tips significantly increased by 95% on Day 3 (the average soil water potential in drying pots was –0.38 MPa), earlier than an increase in ABA concentration in the third leaf of these plants, which increased significant only from Day 4 (Figs 1B, 6A, C; Supplementary Fig. S1). In root tips, soil drying continued to stimulate the ABA concentration on Days 4, 5, and 6, when the concentration was 3, 9, and 12 times of that in well-watered plants, respectively (Fig. 6C). It has to be noted that the root tips were sampled for ABA assay, whereas the entire root system was used for ethylene analysis. From Day 4, the root ethylene release rate in the drying treatment was significantly lower than that of the watered treatment (Fig. 6D). In roots of the well-watered controls, the rate of ethylene release increased by 23–54% on Days 4–6 compared with Day 1 (Fig. 6D).

## Discussion

### Different responses of maize leaf and root growth during soil drying

**Fig. 7.**
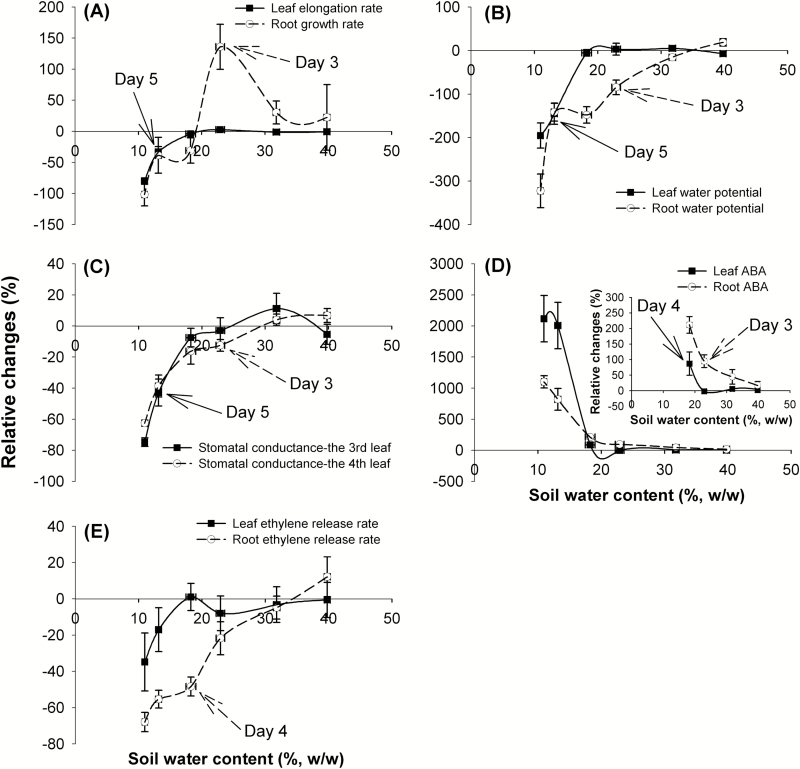
Relative differences in growth and physiology responses of plants exposed to soil drying compared with those that were well-watered during the 6 d experimental period. The relative changes in (A) leaf and root growth rates, (B) leaf and root water potentials, (C) stomatal conductance of the third and fourth leaves, (D) leaf and root ABA concentrations, and (E) ethylene release rates of leaf and root. Points and bars are means ± SEs. Arrows and Day indicate the time when the two treatments became significantly different.

Previous studies have reported that shoot and root growth in maize respond differently during soil drying (Sharp and Davies, 1979; Watts *et al.*, 1981). Shoot growth can be inhibited during soil drying (Sharp and Davies, 1979, 1985; Westgate and Boyer, 1985), while root growth can be stimulated under mild drought and inhibited when the drought becomes severe (Sharp and Davies, 1979; Watts *et al.*, 1981; Creelman *et al.*, 1990). Similarly, in this study, roots of maize plants under the soil drying treatment showed higher growth rates under mild drought (Days 2–3, the average soil water potential in drying pots decreased from –0.31 MPa to –0.38 MPa), but a lower growth rate once the drought became more severe (after Day 3) (Figs 1B, 3, 7A; Supplementary Figs S1, S3). In contrast, leaf elongation was inhibited by soil drying, but only when the drought became more severe, during Days 4–5 (the average soil water potential in drying pots decreased from –0.51 MPa to –0.63 MPa) (Figs 1B, 2, 7A; Supplementary Fig. S1). Modification of shoot and root growth rates can be an important drought avoidance strategy for plants (Lawlor, 2013). Notably, the increase of root growth was the earliest detected developmental change. It has been shown that such stimulation of root growth (especially in deeper soil) under mild drought exerted a positive effect on crop production since it helps maintain water uptake (Manschadi *et al.*, 2006; Kano *et al.*, 2011). However, when the soil volume is limited, or there is little water stored in deep soil layers, there may be little benefit from increased root growth or a deeper root system (Tardieu, 2012; Wasson *et al.*, 2012). Under such conditions, the increased root growth can quickly deplete the small amount of extractable water that remains and then root growth will soon be significantly inhibited (Kamoshita *et al.*, 2004; Tardieu, 2012). Additionally, apart from the severities of drought stress, the plant developmental stages will also affect its shoot and root responses to drought (Boonjung and Fukai, 1996*a*, b; Tardieu, 2012).

In previous studies on maize, roots showed earlier responses to drought (water potential decrease) than shoots (Sharp and Davies, 1979; Westgate and Boyer, 1985; Saab and Sharp, 1989). In the present study, the root water potential started to decrease during Days 2–3 of soil drying (when the average soil water potential in drying pots decreased from –0.31 MPa to –0.38 MPa), while the leaf water potential did not decline until Days 4–5 (when the average soil water potential in drying pots decreased from –0.51 MPa to –0.63 MPa) (Figs 1B, 4A, C, 7B; Supplementary Fig. S1). The later response in the leaf than in the root may be attributable to the early stimulation of root growth under mild drought, allowing the root to take up sufficient water to maintain leaf elongation and leaf water relations for a number of days. In addition, the water potential gradient between leaves and roots/soil was increased during Days 2–3 of soil drying due to a decrease in the water potentials of root and soil, while the leaf water potential was sustained. This result suggests that the root hydraulic conductance was increased by mild soil drying, since the stomatal conductance of the third leaf was maintained (Scoffoni and Sack, 2017). It has also been reported that root proliferation under drought was able to increase whole root system hydraulic conductance and supply more water for transpiration in grape (Alsina *et al.*, 2011).

The decrease in leaf water potential only after the decrease in root and soil water potential supports the view that while leaf water potential can be an indicator of plant water status, it does not always represent the water status of the soil or the root (reviewed in Davies and Zhang, 1991). This is because leaf water potential may not change synchronously with reductions in soil water potential, and other physiological responses may have already been activated in roots and perhaps also in leaves (e.g. reduced stomatal conductance and leaf elongation) (Sharp and Davies, 1979; Bahrun *et al.*, 2002). Some studies suggest that leaf growth inhibition and stomatal closure are the earliest plant responses to drought, and the former is earlier than the latter (Hsiao, 1973; Chaves, 1991; Osório *et al.*, 1998). However, these conclusions are often reached in studies where changes in root growth and physiology are not quantified. It is worthy of note that, to avoid the effect of growth-induced water potential in leaves and roots samples (Cavalieri and Boyer, 1982; Boyer, 2017), growing tissue (e.g. root tips and young leaves) was not used for water potential measurements.

The calculated leaf and root turgor pressures were maintained during the 6 d period of soil drying (Fig. 4B, D), which resulted from a reduced solute potential in tissues through osmotic adjustment. The maintenance of turgor pressure is important for tissue to continue growing despite the decrease of tissue water potential (Boyer, 2017). Interestingly, the root turgor pressure in droughted plants increased from 4 d after the last watering when the soil drying became more severe (Fig. 4D), but this was after the increase in root growth in droughted plants. A similar increase in leaf turgor pressure under drought has been seen in two out of seven pearl millet accessions included in the study of Kusaka *et al.* (2005). This may be an adaptation of plants to maintain tissue growth under soil drying when tissue water potential is reduced.

In this study, stomatal conductance in the third leaf was reduced by soil drying from Day 5 (the average soil water potential in drying pots was –0.63 MPa), when the leaf water potential dropped (Figs 1B, 4A, 5A, 7B, C; Supplementary Fig. S1). This is different from previous reports that stomata can start to close before leaf water potential is reduced by soil drying (Bahrun *et al.*, 2002; Tardieu *et al.*, 2010). Reduced stomatal conductance is a typical drought avoidance strategy in many plant species because it prevents continued high rates of water loss from leaves and thereby postpones or minimizes potential damage by more severe decreases in water potential and turgor (Lawlor, 2013).

Interestingly, in our experiments, the younger leaf (the fourth) showed lower stomatal conductance on Day 3 (the average soil water potential in drying pots was –0.38 MPa) when only the water potential of the root was significantly reduced by soil drying (Figs 1B, 4C, 5B, 7B, C; Supplementary Fig. S1). This could be explained if stomata of the younger leaves were more sensitive to soil drying than those of the older leaves, but there is still a question of how the stomata respond to a change in root water potential while the water potential of the leaves is not affected by soil drying. Stomata of the fourth leaf may be responding to an ABA-based root signal but, if this is the case, why do stomata of the third leaf not respond to this signal? Stomata in older leaves have been found to be less sensitive to ABA than those of relatively younger leaves (Chen *et al.*, 2013). The results also indicate that the stomata of the growing leaf responded more quickly to soil drying than did its elongation rate. Leaf water potential in the fourth leaf was not measured, so it is not clear whether soil drying reduced both the water potential and stomatal conductance in the fourth leaf at the same time or not. Bajji *et al.* (2001) found that the decreases of leaf water potential and solute potential were larger in younger growing leaves than those in relatively older leaves in three wheat cultivars when subjected to the same 15 d progressive soil drying. It was suggested that this phenomenon may be associated with the higher capacity of younger leaves for osmotic adjustment and maintenance of cellular water content and turgor (Morgan, 1984; Bajji *et al.*, 2001). Water potential in younger leaves could also be more depressed than in mature leaves due to possible hydraulic limitation in the growing zone at the base of the younger leaves. If this was the case, such a decrease in leaf water potential of the fourth leaf (younger leaf) (not measured) might have stimulated ABA production here. As highlighted above, intraorgan variation in water status can be a complication in analysis of the kind attempted here (Buckley *et al.*, 2017).

The literature reports that older leaves can provide ABA to sustain higher ABA concentrations in younger leaves (Zeevaart and Boyer, 1984; Chater *et al.*, 2014), but there is no evidence of this here. Thus, these results indicated that earlier root physiological responses to soil drying and stomatal closure in younger leaves may be better indicators to define the onset and severity of a drought event than leaf growth inhibition and other later responses in leaves. Furthermore, stomatal closure in young leaves will be easier to measure than root responses when plants are grown in soil.

### The relationship between the ABA concentration, ethylene release rate, and the leaf and root growth during soil drying

It is often unclear from the literature at which stage plant hormone levels start to change following the initiation of a soil drying episode and whether these changes are synchronous with other root or leaf physiological changes. In this study, it was found that ABA concentrations in both root tips and leaf tissues of maize increased under soil drying (Fig. 6A, C), which is in accordance with previous studies (Davies and Zhang, 1991). Where the extra ABA came from in those samples of droughted plants cannot be determined in this study, but extra ABA is detected in the root before a decline in leaf water potential is detected (although a possible decrease in water status of younger leaves is discussed above). It may be newly synthesized or released from stored inactive glucose ester conjugate either in sampled tissues or circulated from other tissues (Wasilewska *et al.*, 2008). Interestingly, the accumulation of ABA in the roots triggered by soil drying was accompanied by a stimulation of root growth on the same day (Days 2–3, mild drought, the average soil water potential in drying pots decreased from –0.31 MPa to –0.38 MPa), (Figs 1B, 7A, D; Supplementary Fig. S1). After Day 3, as the soil moisture content declined further, ABA continued to accumulate in roots and this was accompanied by slower rates of root growth (Fig. 7A, D). Exogenous ABA has been found to both stimulate and inhibit root growth in maize, rice, and also Arabidopsis, depending on its concentration (Watts *et al.*, 1981; Xu *et al.*, 2013; Li *et al.*, 2017). Therefore, this suggests that increased ABA levels in roots may have either stimulated or inhibited root growth, depending on the magnitude of ABA accumulation under a mild or a more severe drought. In contrast to the root, the ABA concentration in the leaf increased later, during Days 3–4 (Fig. 7D). However, the leaf elongation rate was inhibited later, during Days 4–5 (Fig. 7A). This indicates that a small increase of leaf ABA (~2-fold increase) was not related to a change in leaf elongation rate, while a large increase in leaf ABA level coincided with the inhibition of leaf elongation, which is consistent with previous reports that ABA is an inhibitor of shoot growth (Sharp and LeNoble, 2002; Meguro and Sato, 2014).

In this study, root tips were sampled only from the top two-thirds of the pot to analyse ABA concentration, because the root sampling method can be important if we want to argue that root ABA increase occurred together with the decrease of root water potential. Soil water was distributed heterogeneously in the pot (Fig. 1B), so that when the top part of the soil column is dry enough to trigger an increase of ABA concentration in the root, the lower part may still be too wet to see any enhanced root ABA level. Thus, if root tips are collected from the entire soil column, this may make it difficult to see an early increase of ABA concentration in the root even when the average soil water content had dropped to 22% in a preliminary experiment (data not shown). Puértolas *et al.* (2015) reported a similar finding in potato plants, which were grown in a vertical partial root-zone drying system, that roots sampled in the lower wetter part of a soil column had a lower ABA concentration than roots in the upper, drier soil.

The present study showed that soil drying inhibited ethylene release from both maize leaves and roots (Fig. 6B, D), which is in accordance with the finding that maize ethylene emission was inhibited under low water potentials when the ABA level was increased (Sharp and LeNoble, 2002). However, the inhibitory effects of soil drying on leaf and root ethylene occurred at a later stage of the soil drying than the ABA accumulation (on Day 6 and 4, respectively) (Fig. 7E). Thus, the ABA concentrations in leaf and root were more susceptible to soil drying than ethylene release rates. Furthermore, both the leaf and root growth responses had occurred prior to the detected changes of ethylene level during soil drying (Fig. 7A, E). These non-synchronous effects suggest that changes in ethylene level do not play an important role in the regulation of leaf elongation and root growth under drought (at least before Day 4 in the current experiment). Similarly, Voisin *et al.* (2006) found that the leaf elongation rate was not affected in moderately drought-stressed ABA-deficient maize plants that showed high ethylene levels. One further possibility is that the ethylene emissions may have been affected by the soil drying in the first few days of soil drying, but the GC equipment may not be sufficiently sensitive to detect such small changes (Cristescu *et al.*, 2013).

A possible explanation for the increase in root ethylene levels of well-watered plants from Day 4 is that the container has constrained the growing volume of the root system and caused stress (Poorter *et al.*, 2012) (Fig. 6D). Ethylene has been reported to be a stress-induced hormone. Mechanical impedance can enhance the ethylene production without changing the ABA level, while phosphorus deficiency can also promote ethylene emissions (Moss *et al.*, 1988; Li *et al.*, 2009).

Results from this work indicate when and how the hydraulic and chemical (hormonal) changes in maize leaves and roots could regulate stomatal conductance and plant growth in response to initially very small changes in soil water status during a 6 d non-lethal drying. It is suggested that ABA accumulation may play important roles in regulating both root growth promotion and inhibition during different stages of soil drying, while a reduced ethylene content may not be involved in regulating leaf and root growth at an early stage of drying. These early developmental and physiological responses may be key to crop establishment. However, plants are complex systems, and different results could be seen with different time scales of drought treatments (short term versus long term), plant genotypes, or soil conditions (e.g. soils with different depths) (Tardieu and Parent, 2017). The identification of the critical point at which soil water status affects root growth (either positively or negatively), along with the other observed physiological responses (e.g. stomatal conductance reduction in different leaves and changes in leaf and root water potential) focuses attention of physiological and developmental changes that can influence both agronomy and crop improvement strategies for establishment of crops in dryland environments. It is clear that considerable precision in both chemical and hydraulic status of different plant parts is important if we are to understand which are the controlling influences for growth, development, and functioning of plants under drought.

## Supplementary Data

Supplementary data are available at *JXB* online.

Table S1. Soil water content data from a preliminary 5 d soil drying experiment.

Fig. S1. Soil water characteristic curve: soil water potential against soil water content.

Fig. S2. Leaf elongation rate of the fourth leaf (leaf was fully expanded on Day 2 or 3), the sixth leaf (leaf was expanding and visible from Day 1), and the seventh leaf (leaf was expanding and visible from Day 4).

Fig. S3. Rates of root growth and increase in total root surface area during the 6 d soil drying treatment.

Fig. S4. Leaf ABA concentration and ethylene release rate results from a preliminary 5 d soil drying experiment.

## Supplementary Material

Supplementary Table and Figures

## Abbreviations:

ABA: abscisic acid
CE: controlled-environment.

## References

[CIT0001] Alsina MM, Smart DR, Bauerle T, de Herralde F, Biel C, Stockert C, Negron C, Save R 2011 Seasonal changes of whole root system conductance by a drought-tolerant grape root system. Journal of Experimental Botany 62, 99–109. 20851906 10.1093/jxb/erq247 PMC2993904

[CIT0002] Arraes FB, Beneventi MA, Lisei de Sa ME, Paixao JF, Albuquerque EV, Marin SR, Purgatto E, Nepomuceno AL, Grossi-de-Sa MF 2015 Implications of ethylene biosynthesis and signaling in soybean drought stress tolerance. BMC Plant Biology 15, 213. 26335593 10.1186/s12870-015-0597-z PMC4557918

[CIT0003] Bahrun A, Jensen CR, Asch F, Mogensen VO 2002 Drought-induced changes in xylem pH, ionic composition, and ABA concentration act as early signals in field-grown maize (*Zea mays* L.). Journal of Experimental Botany 53, 251–263. 11807129 10.1093/jexbot/53.367.251

[CIT0004] Bajji M, Lutts S, Kinet J 2001 Water deficit effects on solute contribution to osmotic adjustment as a function of leaf ageing in three durum wheat (*Triticum durum* Desf.) cultivars performing differently in arid conditions. Plant Science 160, 669–681. 11448742 10.1016/s0168-9452(00)00443-x

[CIT0005] Battisti DS, Naylor RL 2009 Historical warnings of future food insecurity with unprecedented seasonal heat. Science 323, 240–244. 19131626 10.1126/science.1164363

[CIT0006] Boonjung H, Fukai S 1996 *a* Effects of soil water deficit at different growth stages on rice growth and yield under upland conditions. 1. Growth during drought. Field Crops Research 48, 37–45.

[CIT0007] Boonjung H, Fukai S 1996 *b* Effects of soil water deficit at different growth stages on rice growth and yield under upland conditions. 2. Phenology, biomass production and yield. Field Crops Research 48, 47–55.

[CIT0008] Boyer J, Byrne P, Cassman K, Cooper M, Delmer D, Greene T, Gruis F, Habben J, Hausmann N, Kenny N 2013 The US drought of 2012 in perspective: a call to action. Global Food Security 2, 139–143.

[CIT0009] Boyer JS 1982 Plant productivity and environment. Science 218, 443–448. 17808529 10.1126/science.218.4571.443

[CIT0010] Boyer JS 2017 Plant water relations: a whirlwind of change. Springer: Heidelberg.

[CIT0011] Brodribb TJ 2009 Xylem hydraulic physiology: the functional backbone of terrestrial plant productivity. Plant Science 177, 245–251.

[CIT0012] Buckley TN, John GP, Scoffoni C, Sack L 2017 The sites of evaporation within leaves. Plant Physiology 173, 1763–1782. 28153921 10.1104/pp.16.01605 PMC5338672

[CIT0013] Cavalieri AJ, Boyer JS 1982 Water potentials induced by growth in soybean hypocotyls. Plant Physiology 69, 492–496. 16662235 10.1104/pp.69.2.492 PMC426236

[CIT0014] Challinor AJ, Watson J, Lobell DB, Howden SM, Smith DR, Chhetri N 2014 A meta-analysis of crop yield under climate change and adaptation. Nature Climate Change 4, 287–291.

[CIT0015] Chater CC, Oliver J, Casson S, Gray JE 2014 Putting the brakes on: abscisic acid as a central environmental regulator of stomatal development. New Phytologist 202, 376–391. 24611444 10.1111/nph.12713

[CIT0016] Chaves MM 1991 Effects of water deficits on carbon assimilation. Journal of Experimental Botany 42, 1–16.

[CIT0017] Chen L, Dodd IC, Davies WJ, Wilkinson S 2013 Ethylene limits abscisic acid- or soil drying-induced stomatal closure in aged wheat leaves. Plant, Cell and Environment 36, 1850–1859. 10.1111/pce.12094 23488478

[CIT0018] Creelman RA, Mason HS, Bensen RJ, Boyer JS, Mullet JE 1990 Water deficit and abscisic acid cause differential inhibition of shoot versus root growth in soybean seedlings: analysis of growth, sugar accumulation, and gene expression. Plant Physiology 92, 205–214. 16667248 10.1104/pp.92.1.205 PMC1062271

[CIT0019] Cristescu SM, Mandon J, Arslanov D, De Pessemier J, Hermans C, Harren FJ 2013 Current methods for detecting ethylene in plants. Annals of Botany 111, 347–360. 23243188 10.1093/aob/mcs259 PMC3579434

[CIT0020] Davies WJ, Zhang JH 1991 Root signals and the regulation of growth and development of plants in drying soil. Annual Review of Plant Physiology and Plant Molecular Biology 42, 55–76.

[CIT0021] Durand M, Porcheron B, Hennion N, Maurousset L, Lemoine R, Pourtau N 2016 Water deficit enhances C export to the roots in *Arabidopsis thaliana* plants with contribution of sucrose transporters in both shoot and roots. Plant Physiology 170, 1460–1479. 26802041 10.1104/pp.15.01926 PMC4775148

[CIT0022] Gilbert ME, Medina V 2016 Drought adaptation mechanisms should guide experimental design. Trends in Plant Science 21, 639–647. 27090148 10.1016/j.tplants.2016.03.003

[CIT0023] Harris JM 2015 Abscisic acid: hidden architect of root system structure. Plants 4, 548–572. 27135341 10.3390/plants4030548 PMC4844405

[CIT0024] Hsiao TC 1973 Plant responses to water stress. Annual Review of Plant Physiology 24, 519–570.

[CIT0025] Huang D, Wu W, Abrams SR, Cutler AJ 2008 The relationship of drought-related gene expression in *Arabidopsis thaliana* to hormonal and environmental factors. Journal of Experimental Botany 59, 2991–3007. 18552355 10.1093/jxb/ern155 PMC2504347

[CIT0026] Kamoshita A, Rodriguez R, Yamauchi A, Wade LJ 2004 Genotypic variation in response of rainfed lowland rice to prolonged drought and rewatering. Plant Production Science 7, 406–420.

[CIT0027] Kano M, Inukai Y, Kitano H, Yamauchi A 2011 Root plasticity as the key root trait for adaptation to various intensities of drought stress in rice. Plant and Soil 342, 117–128.

[CIT0028] Kazan K 2015 Diverse roles of jasmonates and ethylene in abiotic stress tolerance. Trends in Plant Science 20, 219–229. 25731753 10.1016/j.tplants.2015.02.001

[CIT0029] Kusaka M, Lalusin AG, Fujimura T 2005 The maintenance of growth and turgor in pearl millet (*Pennisetum glaucum* [L.] Leeke) cultivars with different root structures and osmo-regulation under drought stress. Plant Science 168, 1–14.

[CIT0030] Lawlor DW 2013 Genetic engineering to improve plant performance under drought: physiological evaluation of achievements, limitations, and possibilities. Journal of Experimental Botany 64, 83–108. 23162116 10.1093/jxb/ers326

[CIT0031] Li X, Chen L, Forde BG, Davies WJ 2017 The biphasic root growth response to abscisic acid in Arabidopsis involves interaction with ethylene and auxin signalling pathways. Frontiers in Plant Science 8, 1–12. 28890725 10.3389/fpls.2017.01493 PMC5574904

[CIT0032] Li YS, Mao XT, Tian QY, Li LH, Zhang WH 2009 Phosphorus deficiency-induced reduction in root hydraulic conductivity in *Medicago falcata* is associated with ethylene production. Environmental and Experimental Botany 67, 172–177.

[CIT0033] Manschadi AM, Christopher J, Devoil P, Hammer GL 2006 The role of root architectural traits in adaptation of wheat to water-limited environments. Functional Plant Biology 33, 823–837. 10.1071/FP06055 32689293

[CIT0034] McDaniel RL, Munster C, Cothren JT 2017 Crop and location specific agricultural drought quantification: part I. Method development. Transactions of the ASABE 60, 721–728.

[CIT0035] Meguro A, Sato Y 2014 Salicylic acid antagonizes abscisic acid inhibition of shoot growth and cell cycle progression in rice. Scientific Reports 4, 4555. 24686568 10.1038/srep04555 PMC3971400

[CIT0036] Morgan JM 1984 Osmoregulation and water stress in higher plants. Annual Review of Plant Physiology 35, 299–319.

[CIT0037] Morgan PW, He CJ, De Greef JA, De Proft MP 1990 Does water deficit stress promote ethylene synthesis by intact plants?Plant Physiology 94, 1616–1624. 16667895 10.1104/pp.94.4.1616 PMC1077429

[CIT0038] Moss GI, Hall KC, Jackson MB 1988 Ethylene and the responses of roots of maize (*Zea mays* L.) to physical impedance. New Phytologist 109, 303–311.

[CIT0039] Muday GK, Rahman A, Binder BM 2012 Auxin and ethylene: collaborators or competitors?Trends in Plant Science 17, 181–195. 22406007 10.1016/j.tplants.2012.02.001

[CIT0040] Munns R, Cramer GR 1996 Is coordination of leaf and root growth mediated by abscisic acid? Opinion. Plant and Soil 185, 33–49.

[CIT0041] Osório J, Osório ML, Chaves MM, Pereira JS 1998 Water deficits are more important in delaying growth than in changing patterns of carbon allocation in *Eucalyptus globulus*. Tree Physiology 18, 363–373. 12651361 10.1093/treephys/18.6.363

[CIT0042] Pierik R, Testerink C 2014 The art of being flexible: how to escape from shade, salt, and drought. Plant Physiology 166, 5–22. 24972713 10.1104/pp.114.239160 PMC4149730

[CIT0043] Pierik R, Tholen D, Poorter H, Visser EJ, Voesenek LA 2006 The Janus face of ethylene: growth inhibition and stimulation. Trends in Plant Science 11, 176–183. 16531097 10.1016/j.tplants.2006.02.006

[CIT0044] Poorter H, Bühler J, van Dusschoten D, Climent J, Postma JA 2012 Pot size matters: a meta-analysis of the effects of rooting volume on plant growth. Functional Plant Biology 39, 839–850. 10.1071/FP12049 32480834

[CIT0045] Puértolas J, Conesa MR, Ballester C, Dodd IC 2015 Local root abscisic acid (ABA) accumulation depends on the spatial distribution of soil moisture in potato: implications for ABA signalling under heterogeneous soil drying. Journal of Experimental Botany 66, 2325–2334. 25547916 10.1093/jxb/eru501 PMC4407650

[CIT0046] Quarrie SA, Whitford PN, Appleford NE, Wang TL, Cook SK, Henson IE, Loveys BR 1988 A monoclonal antibody to (S)-abscisic acid: its characterisation and use in a radioimmunoassay for measuring abscisic acid in crude extracts of cereal and lupin leaves. Planta 173, 330–339. 24226540 10.1007/BF00401020

[CIT0047] Romero P, Botía P, Keller M 2017 Hydraulics and gas exchange recover more rapidly from severe drought stress in small pot-grown grapevines than in field-grown plants. Journal of Plant Physiology 216, 58–73. 28577386 10.1016/j.jplph.2017.05.008

[CIT0048] Saab IN, Sharp RE 1989 Non-hydraulic signals from maize roots in drying soil: inhibition of leaf elongation but not stomatal conductance. Planta 179, 466–474. 24201770 10.1007/BF00397586

[CIT0049] Saab IN, Sharp RE, Pritchard J, Voetberg GS 1990 Increased endogenous abscisic acid maintains primary root growth and inhibits shoot growth of maize seedlings at low water potentials. Plant Physiology 93, 1329–1336. 16667621 10.1104/pp.93.4.1329 PMC1062676

[CIT0050] Santner A, Calderon-Villalobos LI, Estelle M 2009 Plant hormones are versatile chemical regulators of plant growth. Nature Chemical Biology 5, 301–307. 19377456 10.1038/nchembio.165

[CIT0051] Scoffoni C, Sack L, Ort D 2017 The causes and consequences of leaf hydraulic decline with dehydration. Journal of Experimental Botany 68, 4479–4496. 28981777 10.1093/jxb/erx252

[CIT0052] Sharp RE 2002 Interaction with ethylene: changing views on the role of abscisic acid in root and shoot growth responses to water stress. Plant, Cell and Environment 25, 211–222. 10.1046/j.1365-3040.2002.00798.x 11841664

[CIT0053] Sharp RE, Davies WJ 1979 Solute regulation and growth by roots and shoots of water-stressed maize plants. Planta 147, 43–49. 24310893 10.1007/BF00384589

[CIT0054] Sharp RE, Davies WJ 1985 Root growth and water uptake by maize plants in drying soil. Journal of Experimental Botany 36, 1441–1456.

[CIT0055] Sharp RE, LeNoble ME 2002 ABA, ethylene and the control of shoot and root growth under water stress. Journal of Experimental Botany 53, 33–37. 11741038

[CIT0056] Skirycz A, Vandenbroucke K, Clauw P 2011 Survival and growth of Arabidopsis plants given limited water are not equal. Nature Biotechnology 29, 212–214. 10.1038/nbt.1800 21390020

[CIT0057] Tanaka Y, Sano T, Tamaoki M, Nakajima N, Kondo N, Hasezawa S 2005 Ethylene inhibits abscisic acid-induced stomatal closure in Arabidopsis. Plant Physiology 138, 2337–2343. 16024687 10.1104/pp.105.063503 PMC1183419

[CIT0058] Tardieu F 2012 Any trait or trait-related allele can confer drought tolerance: just design the right drought scenario. Journal of Experimental Botany 63, 25–31. 21963615 10.1093/jxb/err269

[CIT0059] Tardieu F, Parent B 2017 Predictable ‘meta-mechanisms’ emerge from feedbacks between transpiration and plant growth and cannot be simply deduced from short-term mechanisms. Plant, Cell and Environment 40, 846–857. 10.1111/pce.12822 27569520

[CIT0060] Tardieu F, Parent B, Simonneau T 2010 Control of leaf growth by abscisic acid: hydraulic or non-hydraulic processes?Plant, Cell and Environment 33, 636–647. 10.1111/j.1365-3040.2009.02091.x 20002334

[CIT0061] Tuberosa R, Salvi S, Giuliani S, Sanguineti MC, Bellotti M, Conti S, Landi P 2007 Genome-wide approaches to investigate and improve maize response to drought. Crop Science 47, S120–S141.

[CIT0062] Varshney RK, Ribaut JM, Buckler ES, Tuberosa R, Rafalski JA, Langridge P 2012 Can genomics boost productivity of orphan crops?Nature Biotechnology 30, 1172–1176. 10.1038/nbt.2440 23222781

[CIT0063] Voisin AS, Reidy B, Parent B, Rolland G, Redondo E, Gerentes D, Tardieu F, Muller B 2006 Are ABA, ethylene or their interaction involved in the response of leaf growth to soil water deficit? An analysis using naturally occurring variation or genetic transformation of ABA production in maize. Plant, Cell and Environment 29, 1829–1840. 10.1111/j.1365-3040.2006.01560.x 16913872

[CIT0064] Wasilewska A, Vlad F, Sirichandra C, Redko Y, Jammes F, Valon C, Frei dit Frey N, Leung J 2008 An update on abscisic acid signaling in plants and more. Molecular Plant 1, 198–217. 19825533 10.1093/mp/ssm022

[CIT0065] Wasson AP, Richards RA, Chatrath R, Misra SC, Prasad SV, Rebetzke GJ, Kirkegaard JA, Christopher J, Watt M 2012 Traits and selection strategies to improve root systems and water uptake in water-limited wheat crops. Journal of Experimental Botany 63, 3485–3498. 22553286 10.1093/jxb/ers111

[CIT0066] Watts S, Rodriguez JL, Evans SE, Davies WJ 1981 Root and shoot growth of plants treated with abscisic acid. Annals of Botany 47, 595–602.

[CIT0067] Westgate ME, Boyer JS 1985 Osmotic adjustment and the inhibition of leaf, root, stem and silk growth at low water potentials in maize. Planta 164, 540–549. 24248230 10.1007/BF00395973

[CIT0068] Wilhite DA, Glantz MH 1985 Understanding the drought phenomenon: the role of definitions. Water International 10, 111–120.

[CIT0069] Xu W, Jia L, Shi W, Liang J, Zhou F, Li Q, Zhang J 2013 Abscisic acid accumulation modulates auxin transport in the root tip to enhance proton secretion for maintaining root growth under moderate water stress. New Phytologist 197, 139–150. 23106247 10.1111/nph.12004

[CIT0070] Zeevaart JA, Boyer GL 1984 Accumulation and transport of abscisic acid and its metabolites in *Ricinus* and *Xanthium*. Plant Physiology 74, 934–939. 16663536 10.1104/pp.74.4.934 PMC1066795

[CIT0071] Zhang JH, Davies WJ 1989 Sequential response of whole plant water relations to prolonged soil drying and the involvement of xylem sap ABA in the regulation of stomatal behavior of sunflower plants. New Phytologist 113, 167–174.

[CIT0072] Zhang S, Zhang L, Zhou K, Li Y, Zhao Z 2017 Changes in protein profile of *Platycladus orientalis* (L.) roots and leaves in response to drought stress. Tree Genetics and Genomes 13, 76.

