# Supplementary material for: Stomatal and growth responses to hydraulic and chemical changes induced by progressive soil drying
Source: J Exp Bot. 2017 Nov 6;68(21-22):5883–94. doi: 10.1093/jxb/erx381 (PMC5854116; doi:10.1093/jxb/erx381)
Supplement: Supplementary Table and Figures [file erx381_suppl_supplementary_table_s1_figures_s1_s4.pdf]

Supplementary Data Table S1: Soil water content data (means ± standard errors) from a preliminary 5-d soil drying experiment. The growth conditions were the same as the experiments in the main text.

**Days after last watering**	**Soil water content (%, w/w)**	
	**Well-watered**	**Soil drying**
1	42 ± 2	40 ± 1
2	40 ± 2	33 ± 0
3	40 ± 1	22 ± 1
4	40 ± 1	16 ± 1
5	36 ± 1	12 ± 1

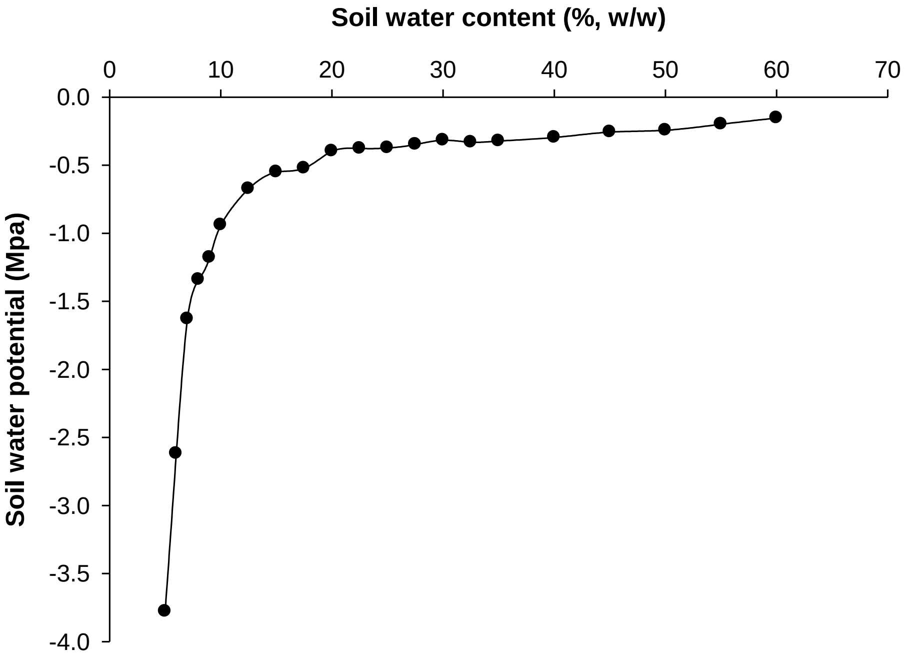

Supplementary Data Figure S1: Soil water characteristic curve: soil water potential against soil water content. (John Innes No.2, Foremost, UK).

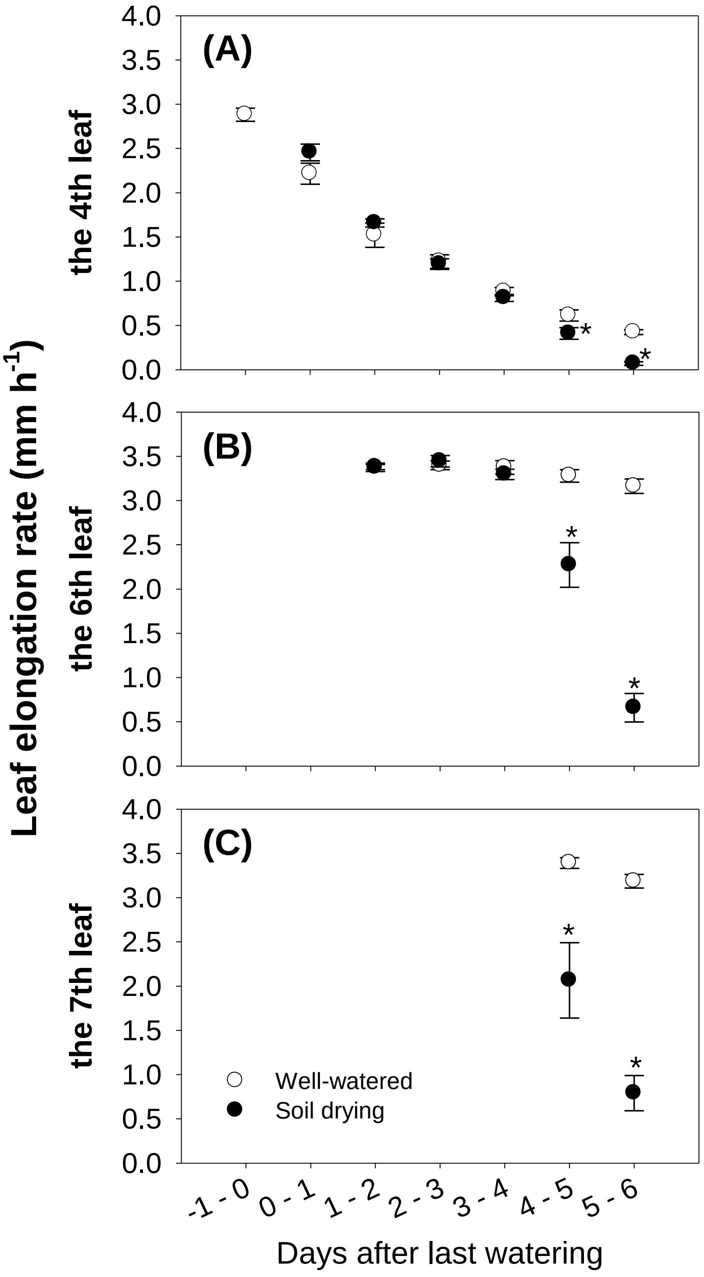

Supplementary Data Figure S2: Leaf elongation rate of (A) the 4th leaf (leaf was fully expanded on Day 2 or 3), (B) the 6th leaf (leaf was expanding and visible from Day 1), (C) the 7th leaf (leaf was expanding and visible from Day 4). Replication n = 7–13. Points and bars are means ± standard errors. Data was analysed using *t*-test and stars indicate significant difference between well-watered and soil drying treatments on the same day at $P < 0.05$.

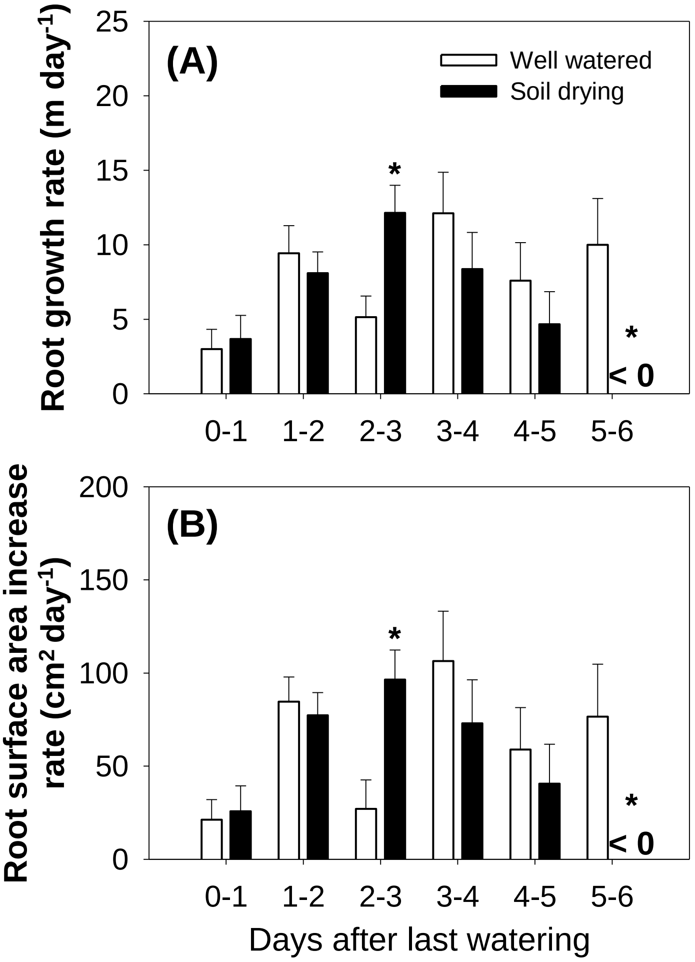

Supplementary Data Figure S3: (A) Root growth rate, (B) total root surface area increase rate during the 6-day soil drying treatment. Columns and bars are means ± standard errors. Data was analysed using *t*-test and stars indicate significant difference between well-watered and soil drying treatments on the same day at $P < 0.05$ (n = 9).

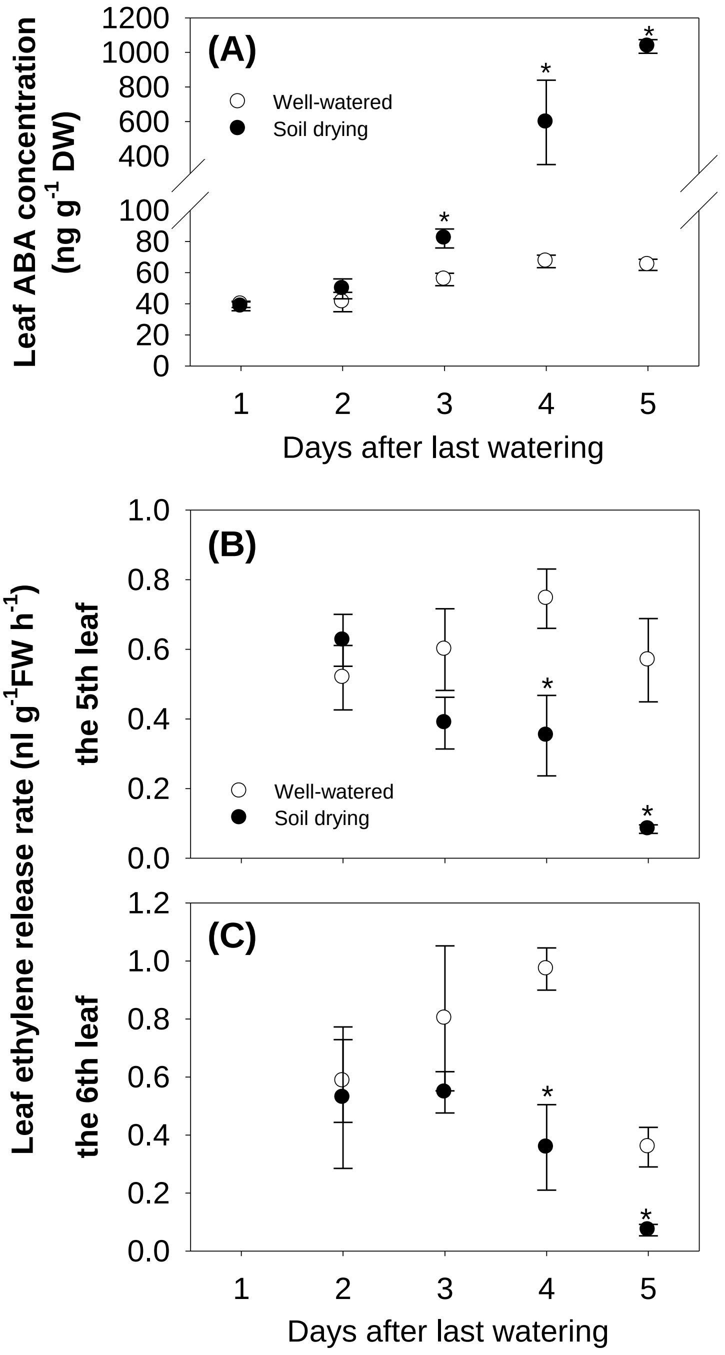

Supplementary Data Figure S4: Leaf ABA concentration and ethylene release rate results from a preliminary 5-d soil drying experiment. (A) Leaf ABA concentration (the 3rd leaf). The leaf ethylene release rate (nl g^{-1} FW h^{-1}) of (B) the 5th leaf, (C) the 6th leaf. Soil water content data can be found in Supplementary Data Table S1. Points and bars are means ± standard errors. Data was analysed using *t*-test and stars indicate significant difference between well-watered and soil drying treatments on the same day at $P < 0.05$ (n = 4).
